# The anti-cancer components of *Ganoderma lucidum* possesses cardiovascular protective effect by regulating circular RNA expression

**DOI:** 10.18632/oncoscience.316

**Published:** 2016-08-28

**Authors:** Yi-Zhen Xie, Fenghua Yang, Weijiang Tan, Xiangmin Li, Chunwei Jiao, Ren Huang, Burton B. Yang

**Affiliations:** ^1^ State Key Laboratory of Applied Microbiology Southern China, Guangdong Institute of Microbiology, Guangzhou, China; ^2^ Guangdong Yuewei Edible Fungi Technology Co. Ltd., Guangzhou, China; ^3^ Guangdong Laboratory Animals Monitoring Institute, Guangdong Provincial Key Laboratory of Laboratory Animals, Guangzhou, Guangdong, China; ^4^ Sunnybrook Research Institute, Sunnybrook Health Sciences Centre, Toronto, ON, Canada; ^5^ Department of Laboratory Medicine and Pathobiology, University of Toronto, Toronto, ON, Canada

**Keywords:** herbal medicine, Ganoderma lucidum, anti-cancer, heart function, circular RNA

## Abstract

To examine the role of oral *Ganoderma* spore oil in cardiovascular disease, we used transverse aortic constriction (TAC) in mice to model pressure overload-induced cardiomyopathy. Our preliminary results demonstrated a potential cardioprotective role for spore oil extracted from *Ganoderma*. We found that *Ganoderma* treatment normalized ejection fraction and corrected the fractional shortening generated by TAC. We also found evidence of reduced left ventricular hypertrophy as assessed by left ventricular end diastolic diameter. Analysis of total RNA expression using cardiac tissue samples from these mice corroborated our findings. We found reduced expression of genes associated with heart failure, including a novel circular RNA circ-Foxo3. Thus our data provides evidence for *Ganoderma lucidum* as a potential cardioprotective agent, warranting further preclinical exploration.

## INTRODUCTION

Complementary and alternative medicines have attracted increasing attention as disease treatments, adjuvants, and alternative supplements [1–4]. Medicinal mushrooms comprise a large proportion of these alternative products, among which Ganoderma lucidum is the most highly studied [5–8]. Preclinical studies have demonstrated anti-tumorigenic roles in a range of medicinal mushrooms [9–11]. A Cochrane meta- analysis showed that patients who had been administered Ganoderma lucidum alongside chemo/radiotherapy were more likely to respond positively compared to chemo/radiotherapy alone. These trials demonstrated improved immune functions as measured by increased CD3, CD4, and CD8 immune response cells [12]. In vitro, Ganoderma lucidum was also found to inhibit proliferation and induce apoptosis in ovarian, colon, and gastric cancer cell lines [13–15]. Ganoderma lucidum contains beta glucans and other polysaccharides which stimulate innate immunity and activate host dendritic cells [16, 17]. Ganoderma lucidum also produces a group of ganoderic acids, which have molecular structures that are similar to steroids [18, 19].

## DISCUSSION

An exploratory trial of 26 patients with hypertension and/or dyslipidemia demonstrated that *Ganoderma lucidum* treatment reduced total triglycerides and increased HDL-cholesterol, implicating a cardio-protective role [20]. We employed a transverse aortic constriction (TAC) mouse model of pressure overload-induced cardiomyopathy and heart failure to examine the role of Ganoderma spore oil administration. The TAC model induces an initial compensatory cardiac remodeling which enhances cardiac contractility. Gradually, the response to chronic overload leads to cardiac dilatation and heart failure. The murine TAC model has been extensively used to study cardiovascular disease and to elucidate signaling pathways involved in cardiac hypertrophy and heart failure.

TAC mice were administered oral Ganoderma spore oil every other day for 14 days. The control group were administered vegetable oil and an anti-hypertensive β2 adrenergic receptor antagonist. Mice were anesthetized with 2% isoflurane inhalation for transthoracic echocardiography and invasive hemodynamic assessment. Transthoracic echocardiography was performed to measure left ventricular ejection fraction (LVEF), left ventricular fractional shortening (LVFS), left ventricular end diastolic diameter, and cardiac output. Data analysis was conducted in a blinded manner.

**Fig 1:**
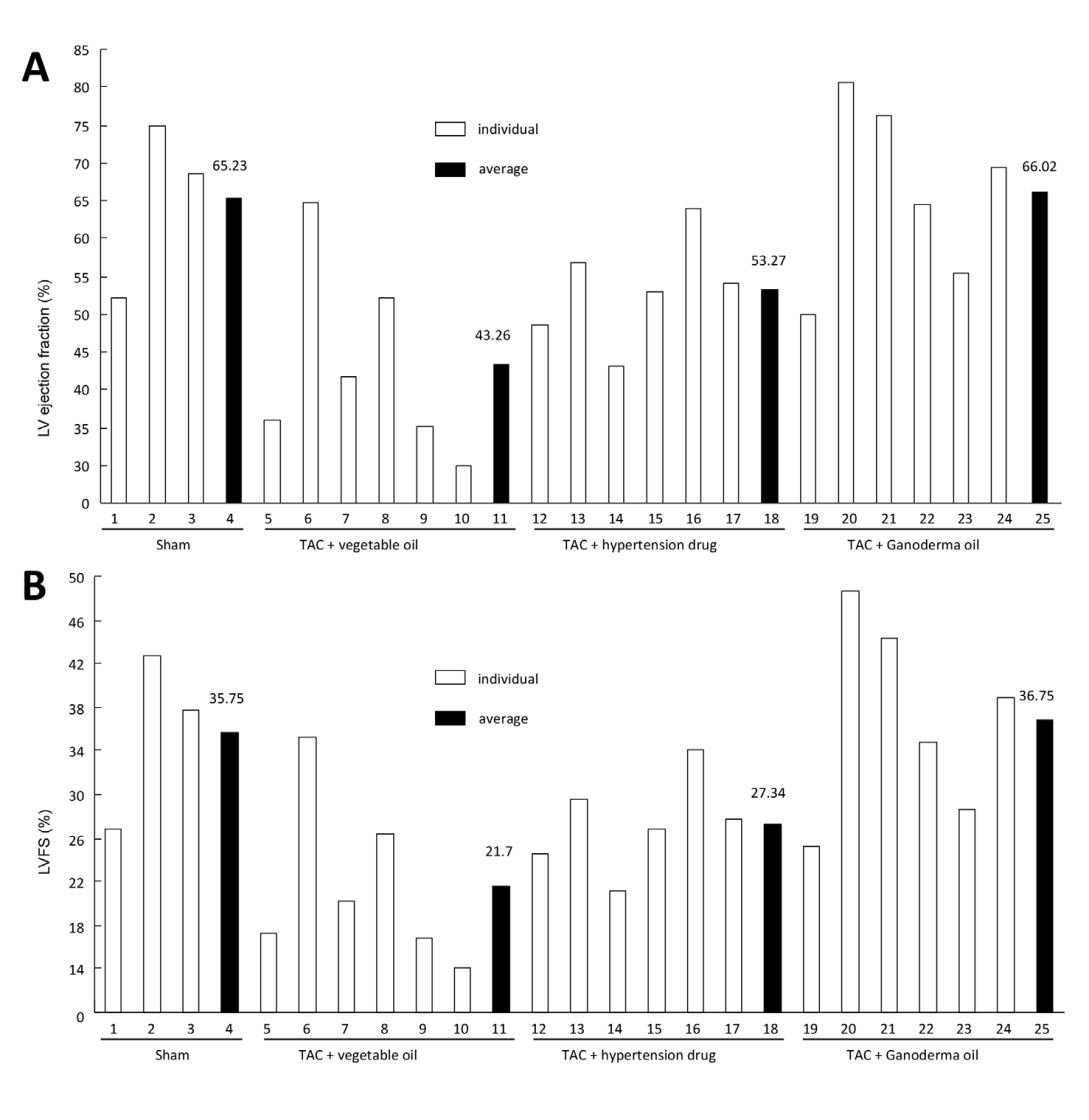
Ganoderma spore oil improves ejection fraction and fractional shortening TAC mice showed decreased levels of LVEF (A) and LVFS (B). Treatment with Ganoderma spore oil increased the levels of LVEF and LVFS.

**Fig 2:**
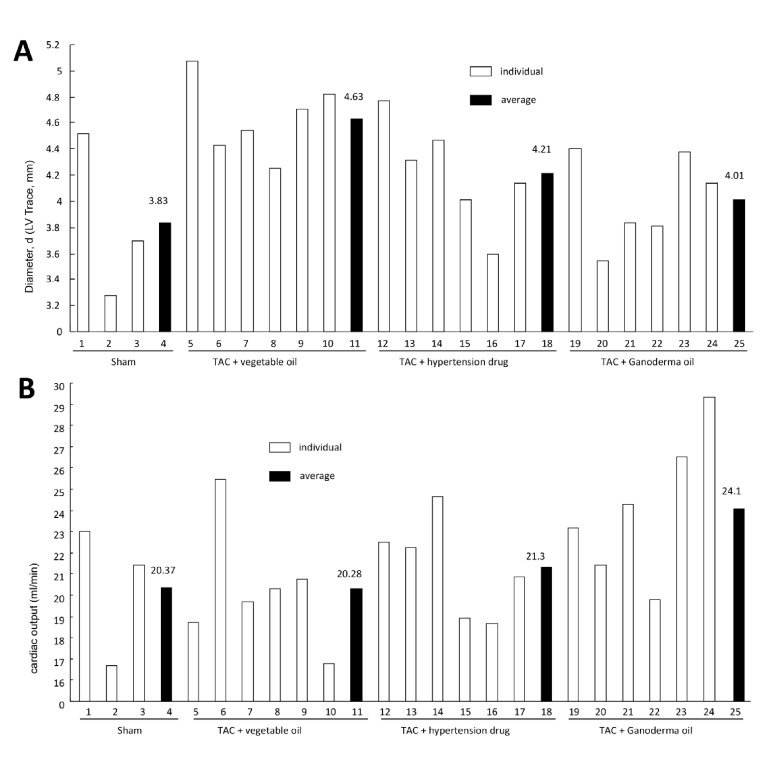
Ganoderma spore oil improves cardiac output (**A**) TAC mice had increased left ventricular end diastolic diameters, while treatment with Ganoderma spore oil decreased the end diastolic diameters. (**B**) Treatment with Ganoderma spore oil increased cardiac output.

Relative to the 65.23% ejection fraction in healthy sham mice, TAC mice were found to have a 43.26% ejection fraction (Fig 1A). This was below the normal range of 55-75%, validating the TAC model we employed. Treatment with the anti-hypertensive medication approximated the normal range, while delivery of Ganoderma spore oil recovered the stroke volume to normal ranges. Consistent with these results, TAC mice displayed 21.7% fractional shortening (Fig 1B). This was within the mildly abnormal range of 20-25%, while treatment with Ganoderma spore oil brought it to the normal range of 25-45%. The TAC mice also showed increased left ventricular end diastolic diameters, while mice treated with Ganoderma spore oil did not exhibit the same levels of left ventricular hypertrophy (Fig 2A). As a result, mice treated with Ganoderma spore oil recovered to physiologic cardiac output levels at 24.1 ml/min (Fig 2B). This led to improved vascular perfusion within the mice (Fig 2B). This suggests that Ganoderma spore oil increases the heart function to meet the demands of the body.

**Fig 3:**
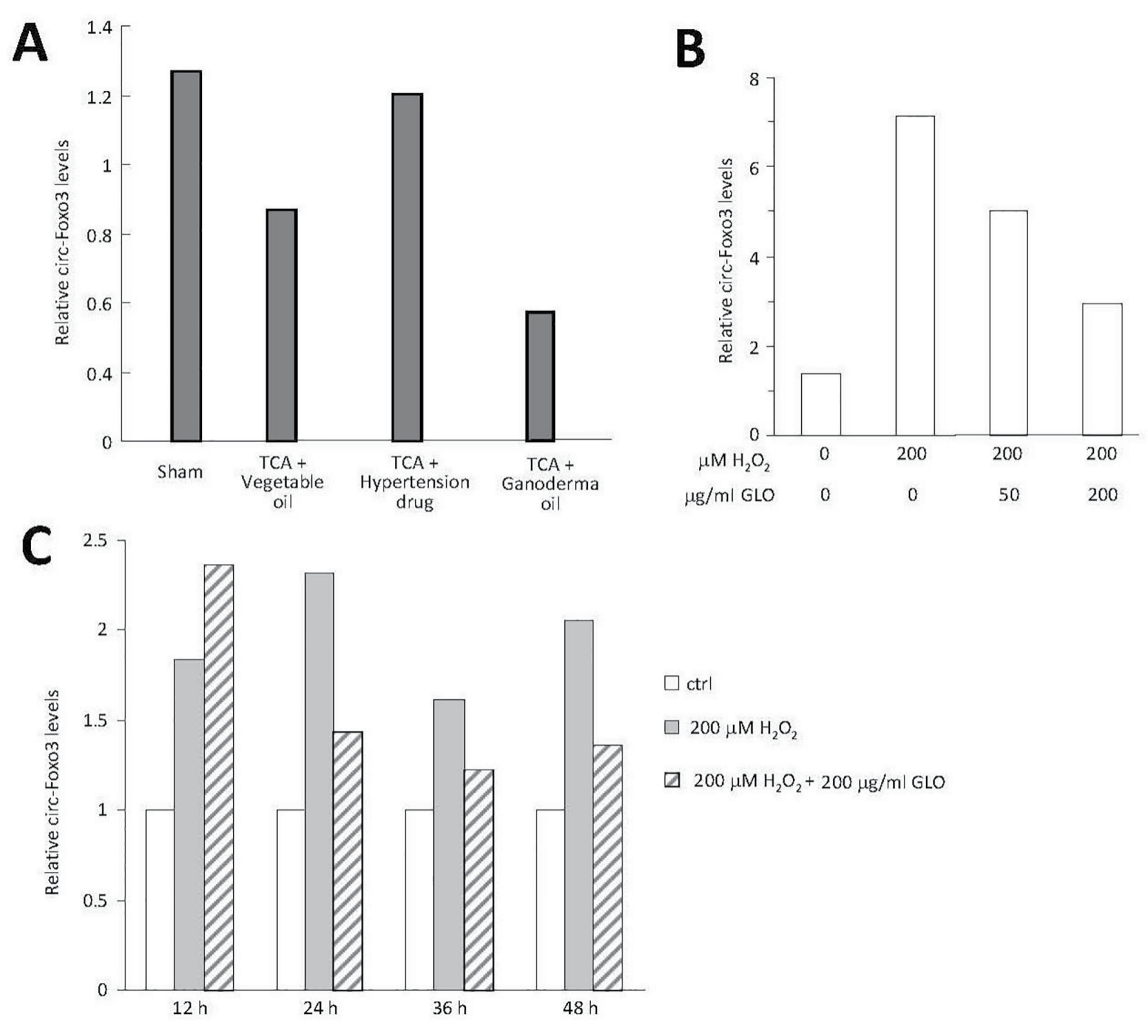
Treatment with Ganoderma spore oil decreases expression of circ-Foxo3 (**A**) Mice orally delivered with Ganoderma spore oil expressed decreased levels of circ-Foxo3 in the heart tissues. (**B**) Mouse cardiac fibroblasts treated with different concentrations of Ganoderma spore oil (GSO) expressed decreased levels of circ-Foxo3 in a concentration-dependent manner. (**C**) Mouse cardiac fibroblasts treated with Ganoderma spore oil expressed lower levels of circ-Foxo3 than the controls, which was time-dependent.

After functional analyses, mice were sacrificed and heart tissue was harvested. Total RNA was extracted with an RNeasy Mini Kit (Qiagen), followed by real-time PCR measurement with miScriptSYBR GreenPCR Kit (Qiagen) as described [21] to analyze levels of a circular RNA circ-Foxo3. We recently demonstrated that expression of circ- Foxo3 RNA could inhibit tumor cell cycle progression [22, 23] and promote cardiac senescence [24]. In this study, mice injected with the chemotherapeutic agent Doxorubicin (Dox) for induction of cardiomyopathy were analyzed for RNA expression levels. Mice with reduced cardiac function had increased expression of circ-Foxo3 RNA [24]. In the current study, we found that treatment with *Ganoderma* spore oil decreased levels of circ-Foxo3 (Fig 3A).

To corroborate these results, we cultured mouse cardiac fibroblasts and treated the cells with *Ganoderma* spore oil, following hydrogen peroxide induced oxidative stress. Relative to control groups, treatment with Ganoderma spore oil decreased circ-Foxo3 levels in a concentration- (Fig 3B) and time-dependent manner (Fig 3C).

Our previous studies have shown that the oil fraction of Ganoderma spores could induce death in versican-transformed cancer cells [25]. Further study found that the Ganoderma spore oil could induce death of cancer stem-like cells [6], potentially mediated by the molecule ergosterol peroxide [19]. Our preclinical results here show that Ganoderma spore oil has a protective role within the cardiovascular system. Treatment in TAC mice was found to normalize ejection fraction and correct the fractional shortening generated by this model. We also found evidence of reduced left ventricular hypertrophy as assessed by left ventricular end diastolic diameter. Thus in our TAC model, cardiac output was improved by oral administration of Ganoderma spore oil. This data provides rationale for further preclinical exploration of Ganoderma lucidum as a cardioprotective agent.
